# An analysis of the contributions of the Collaborations for Leadership in Applied Health Research and Care to the impact of research in the UK Research Excellence Framework 2014 and 2021

**DOI:** 10.1186/s12913-026-14048-6

**Published:** 2026-03-26

**Authors:** Surinder Bangar, Sue Mawson, Jo Cooke

**Affiliations:** 1 Sheffield Centre for Health and Related Research, The University of Sheffield, Regent Court, 30 Regent Street, Sheffield, S1 4DA UK; 2 School of Allied Health Professions, Nursing and Midwifery, The University of Sheffield, 362 Mushroom Lane, Sheffield, S10 2TS UK

**Keywords:** Impact, Research Excellence Framework, Knowledge mobilisation, CLAHRC, Health Research

## Abstract

**Background:**

The National Institute for Health and Care Research (NIHR) established nine pilot Collaborations for Leadership in Applied Health Research and Care (CLAHRC) in 2008 which have since grown to the current fifteen Applied Research Collaborations (ARC). The CLAHRC partnership model focuses upon developing and conducting applied health research to improve outcomes for patients. We explored the scale and focus of the CLAHRCs’ contribution to impact case studies submitted to the UK Research Excellence Framework (REF) 2014 and 2021 and how this might be of value for future health research and practice.

**Methods:**

The study design used a content analysis of the REF 2014 and REF 2021 datasets of impact case studies. We identified impact case studies linked to CLAHRCs from both REF datasets and extracted primarily quantitative data about the institutions involved, subject areas, how they were linked to a CLAHRC and what role the CLAHRC contributed. Qualitative analysis consisted of a purposive sample of eight impact case studies to exemplify the range of subject areas, the scope of involvement of the CLAHRCs and to identify the features associated with the impact.

**Results:**

A total of 53 impact case studies related to a CLAHRC were identified from the REF datasets, an increase from 17 in 2014 to 36 in 2021. There is considerable variation in how CLAHRC involvement is described and making a direct attribution of the CLAHRCs is complex and multifaceted. Key pillars of the CLAHRCs such as undertaking applied health research which responds to local and regional needs, knowledge mobilisation and implementing research into practice are exemplified in the sample of impact case studies. There are multiple mechanisms associated with impact with the benefits of collaborative and partnership activities evident.

**Conclusions:**

Applied research collaborations such as the CLAHRCs are making a significant contribution to the impact of research in the REF. There is scope to improve the visibility of the CLAHRCs and for CLAHRCs/ARCs to provide a valuable model of partnership capable of strengthening the impact of future impact case studies for a range of purposes.

**Supplementary information:**

The online version contains supplementary material available at 10.1186/s12913-026-14048-6.

## Background

It is internationally recognised that Health Services Research should have an impact on society and be good value for money. Societal impact includes improvements in health and wellbeing, and economic gain [1, 2]. An emerging area of exploration is the notion that close partnerships between end users of research, that is health services and patients, and academics promotes research use and impact by working together in partnerships [3]. This paper focuses upon reviewing such partnerships and their research impact.

The group of partnerships under review here were funded by the English National Institute for Health and Care Research (NIHR) with the explicit aim to promote partnership working and coproduction of research, as well as promoting research use [4]. These collaborations were called the Collaborations for Leadership in Applied Health Research and Care (CLAHRCs) and were funded over three consecutive waves by the National Institute for Health and Care Research, each wave lasting over five years with expansion between each one. The first wave included nine CLAHRC pilots, which ran between 2008-2013; the second expanded to include thirteen partnerships between 2013-2019. These first two waves included funding primary research as well as the implementation of research into practice and required the health service partners to provide matched funding, either as time or finance. The third wave of collaborations building from the CLAHRCs were called Applied Research Collaborations (ARCs). These expanded to fifteen partnerships (2019-current). Funding for implementation was not included in this third phase. Appendix 1 provides a list of the Collaborations for Leadership in Applied Health Research and Care/Applied Research Collaborations from 2008-current.

There is debate about how to measure research impact [5–9]. However, during the time when the CLAHRCs/Applied Research Collaborations were funded there was a national approach in the United Kingdom (UK) to assessing research quality and impact. In the UK this is called the Research Excellence Framework (REF). Internationally, there are country-specific systems in place to assess the quality of research with a growing commitment to assessing the impact of research at scale [6, 9]. The REF is a peer assessment of the quality of research undertaken by universities in the UK. The REF is conducted by the four higher education funding bodies collaboratively. The main purposes of the REF are to provide accountability and produce evidence of the benefits of public investment in research, to provide benchmarking information and establish reputational yardsticks [10].

Panels of experts are appointed to assess the submissions, and they produce an overall quality profile for each submission [11, 12]. The quality profile consists of an assessment of the research outputs, research impact and the research environment. Submissions were made to subject-based Units of Assessment (UoAs) within four Panel areas. The four Panels were: Panel A (medicine, health and life sciences), Panel B (physical sciences, engineering and mathematics), Panel C (social sciences) and Panel D (arts and humanities). The REF takes place periodically. During the lifetime of the CLAHRCs/Applied Research Collaborations the REF submissions were at the time points of 2014 and 2021. The outcomes of the REF are used to inform the allocation of around £2 billion p.a. of public funding for research in the university sector [10].

Impact was introduced into the REF for the first time in 2014 and was defined as ‘any effect on, change or benefit to the economy, society, culture, public policy or services, health, the environment or quality of life, beyond academia’. Impact was assessed primarily through the submission of impact case studies and on their ‘reach’ and ‘significance’: reach (spread or breadth of influence on the relevant constituencies), and significance (intensity, influence or effort). A rating system was used to assess each impact case study spanning 4* (outstanding) to 0 (unclassified) [11, 12].

Impact case studies were presented as short documents describing the impact of research beyond academia. The documents followed a prescribed template and included the following sections: the title of the case study, a summary of the impact, a description of the underpinning research, references to the research, details of the impact, and sources of evidence to corroborate the impact claimed [11, 12]. The impact domains in each case study were categorised as either cultural, health, economic, environmental, legal, political, societal or technological [13, 14].

The weighting attached to impact has grown in each successive REF period, forming 25% in 2021 of the overall assessment of research quality, an increase from 20% in 2014. Impact has been assessed highly overall, across all subject areas with 88% of case studies achieving 4*/3* level in 2021, an increase from 84% in 2014 [15, 16]. The main features and requirements for impact case studies for REF submissions in 2014 and 2021 are summarised in Table 1 [11, 12]. This paper examines the CLAHRCs/Applied Research Collaborations footprint of impact using the REF.

**Table 1:** Impact in the UK's Research Excellence Framework

	REF 2014	REF 2021
Assessment components for the overall quality profile for each institution	Outputs: 65% Impact*: 20% Environment: 15% *consisting of 16% for the impact case studies and 4% for the impact template which described the strategy and plans for impact [11]	Outputs: 60% Impact*: 25% Environment: 15% *consisting of 25% for the impact case studies. The impact template is incorporated into the environment section [12]
Definition of impact	‘any effect on, change or benefit to the economy, society, culture, public policy or services, health, the environment or quality of life, beyond academia’ [11]	As for REF 2014 [12]
Assessment of impact	Impact case studies were assessed on their ‘reach’ and ‘significance’ and assigned a level between unclassified to 1– 4 star Reach - ‘the spread or breadth of influence or effect on the relevant constituencies’ and Significance – ‘the intensity or the influence or effort’ [11]	As for REF 2014 [12]
Key timescales for the research and the impact	Timescale for underpinning research: 1 January 1993–31 December 2013 Timescale for demonstrating impact: 2008–2013	Timescale for underpinning research: 1 January 2000–31 December 2020 Timescale for demonstrating impact: 2013–2020
Components of an impact case study	The impact case studies were up to four-page documents which included the following: *Title of the case study* *Summary of the impact* *A description of the underpinning research* *References to the research* *Details of the impact* *Sources to corroborate the impact* [11]	The impact case studies used the same headings as for REF 2014 with additional contextual information provided [12].
Number of case studies per institution	A minimum of two for the submission, with the number overall determined by the number of full-time equivalent staff submitted by the institution.	As for REF 2014 [12]
Number of submissions	154 institutions in 36 subject based units of assessment	154 institutions in 34 subject based units of assessment
Number of impact case studies submitted	6,975	6,791
Number of impact case studies on the impact case study databases	6,679	6,361

The impact case studies have been acknowledged as providing a unique and invaluable source of information about the impact of UK research [17, 18] and could be a means of assessing the types of impact that research partnerships can produce. The impact case studies are available in the public domain as searchable databases [13, 14]. The databases are published under a Creative Commons BY 4.0 International License to enable sharing and use within the license [19]. The publicly available database of impact case studies led to a series of analyses examining the range and features of the impact case studies.

The REF 2014 database contains 6,679 case studies (apart from 296 which had full or partial redactions). The King’s College London and Digital Science report (2015) commissioned to undertake an analysis of REF 2014 impact indicates that UK higher education research has wide and varied benefits on the economy, society, culture, policy, health, the environment and quality of life [17]. The impacts arising from health-related funding are also highlighted in commissioned reports [20, 21]. Existing analyses comment that techniques need to be developed to further probe the impact case studies and reference is made to potentially underestimating the impacts attributable to the National Institute for Health and Care Research funded research [17, 21].

Greenhalgh and Fahy’s (2015) analysis of the REF 2014 impact case studies across the community-based health sciences indicates that impact is mainly described in linear, direct impacts and that these specifically have implications for research collaborations such as the CLAHRCs which are built on non-linear models of impact and suggests that those involved in participatory (co-production) models may have been discouraged from submitting case studies [22]. Kislov et al.’s (2018) systematic review of learning from evaluations of the CLAHRCs notes ‘the relative lack of data about the early impact of CLAHRCs on health care provision or outcomes’ [23]. Whilst the funded evaluations that constituted most of the evidence examined by Kislov et al. (2018) were not primarily focused on collating evidence about impact, at least one of them noted in its abstract as early as 2013 that ‘a growing number of completed projects had demonstrated an impact on clinical practice’ [24].

The National Institute for Health and Care Research impact synthesis report (2016) and CLAHRC's Making A Difference (2015) reports collate examples of impacts arising from health research to provide evidence of impacts and benefits [20, 25]. Whilst the diversity and responsiveness to key priorities across regional communities suggests that the CLAHRCs are not one thing, the national CLAHRC model is associated with co-production across multi-stakeholders, evidence informed implementation of research into practice, together with a collaborative match funding model [23].

To-date there has been no specific analysis to examine the contributions of the CLAHRCs across the REF 2014 and 2021 impact case studies. Whilst previous research is likely to have encompassed some of the case studies associated with a CLAHRC/ARC, they have been broader in scope, with different aims and objectives and with some differences in methodologies used [17, 21, 22]. Prior studies have highlighted the role of partnerships and collaborations as a part of the impact [17, 21, 22]. The CLAHRCs/ARCs are a partnership model, therefore a focus upon the CLAHRCs/ARCs offers a lens to illuminate how these can contribute towards research impact. In this study we set out to undertake an analysis of the contributions of the CLAHRCs to the impact of research in the REF impact case studies. Specifically, the aims of the study were: i) to identify the scope and breadth of impact case studies linked to CLAHRCs in the REF 2014 and REF 2021 datasets; ii) to determine what role CLAHRCs have played and iii) to describe characteristics associated with the identified case studies.

## Methods

The study design used a content analysis of the REF 2014 and REF 2021 datasets of impact case studies. The Research Ethics Committee at the Sheffield Centre for Health and Related Research (formerly the School of Health and Related Research), University of Sheffield approved the study in April 2017 (Ethics application number: 013612) with further modifications to update the study to take account of the REF 2021 dataset in February 2023. Informed consent was not required because all documents were in the public domain. The REF impact case studies databases for both 2014 and 2021 [13, 14] include all the impact case studies for each REF cycle apart from those that Universities decided to redact, for example where they may have contained confidential information.

The study consisted of two phases consisting of a keyword examination of the datasets to identify impact case studies related to the CLAHRCs. This was followed in phase two with an in-depth analysis of impact case studies using a proforma. A preliminary wider review of relevant literature and reports was undertaken to inform the design of the content analysis.

In phase one for the data extraction, the initial starting point was to interrogate the REF dataset to find out which case studies specifically mention CLAHRCs. A search of the REF online dataset using the following search terms was undertaken, “Collaboration for Leadership in Applied Health Research and Care” AND/OR “CLAHRC”. In addition for the REF 2021 dataset, we searched by “Applied Research Collaboration” AND/OR “NIHR ARC” to take account of the evolution of the CLAHRCs to the more recently established Applied Research Collaborations. All searches were carried out at least twice.

As funder information was not always clear; in addition to REF dataset searches we used a range of approaches to verify the link to each CLAHRC. As the database searches relied on CLAHRCs being mentioned in the case study they may not have located all relevant case studies. Therefore, for the REF 2014 dataset contact was made with all CLAHRCs to verify whether the identified list of case studies encompassed all those that they were involved with to ensure that the analysis included all relevant impact case studies. Cross-checking with other impact synthesis reports to ensure that all relevant case studies were included also took place [20, 21]. We did not check the links in the case studies, such as the papers referred to in the references section for the 2014 set as these were verified directly with the CLAHRCs.

Developments to the REF 2021 dataset included information about funders. In 2021, for the identified case studies there was a reference to a CLAHRC/Applied Research Collaboration in all the case studies, therefore we drew upon this information and any other related links, such as checking of papers in the references section for those case studies where it was unclear which specific CLAHRCs/Applied Research Collaborations were involved to establish which case studies were linked to specific CLAHRCs/Applied Research Collaborations. The inclusion criteria consisted of impact case studies which mention CLAHRCs and those impact case studies which were submitted to REF which have a verifiable link to a CLAHRC. We excluded those impact case studies which were not related to CLAHRCs. SB, SM and JC discussed identified case studies using the inclusion criteria and agreed those that should be included in the analysis.

In phase two, we undertook in-depth analysis of impact case studies. SB read the identified case studies. A proforma was designed to extract data and record characteristics associated with the case studies. Appendix 2 provides information about the proforma. The proforma was developed by SB, SM and JC prior to carrying out the analysis and drew upon other published analyses of the REF datasets [17, 21, 22, 26] and was tailored to the CLAHRC context. An initial pilot of the proforma with three case studies was carried out and modifications required were made after the pilot. The piloting phase led to adding an additional area to include specifically which section of a case study that a CLAHRC is referred to. SB re-read all case studies.

The proforma was used to record key characteristics of the case studies and to identify the contributions of CLAHRCs. A Microsoft Excel spreadsheet was used to code details from the case studies. Some areas of the proforma were directly coded following the descriptions in the case studies, for example, submitting institution, whether a CLAHRC is directly mentioned or not. Other areas required judgements to be made, for example, characteristics associated with the pathway to impact, and identifying whether any actionable tools had been generated to support use in practice [27]. For illustrative purposes, we selected a purposive sample of eight impact case studies to highlight the range of disciplinary areas related to CLAHRCs, the on-going links with CLAHRCs, the types of CLAHRC involvement and to identify features associated with the impact.

Random sampling of 20% (*n* = 3) of the REF 2014 case studies was undertaken by JC to check SB's interpretations of the data. All case studies in the REF 2021 dataset were discussed between SB and SM. In addition, any specific queries were also discussed with either JC or SM. Differences and queries were resolved through discussion, and this took place as required through the coding phase.

Data synthesis consisted of bringing together quantitative data, such as identification of the number of case studies linked to a CLAHRC/ARC, the higher education institutions involved, which subject areas of the REF these were submitted to, the funders involved and the categories of impact. Qualitative details requiring interpretation and judgement were recorded onto the proforma and synthesised through identifying emergent patterns and themes. These included identifying characteristics associated with the pathway to impact, the development of actionable tools and the contribution of the CLAHRCs/ARCs to the case study. Themes were initially categorised by SB with discussion with SM and JC to ensure that these were appropriate and to arrive at consensus and agreement. An interim report about the REF 2014 dataset was presented by SM and JC at a national meeting of the CLAHRC Directors and Programme Managers in September 2017.

## Results

Data was extracted to identify case studies related to a CLAHRC, characteristics associated with the stated impacts and the role and contributions of the CLAHRCs. On-going discussions about coding confirmed reliability in interpretations. Queries for discussion focused on clarifying the research and impact timescale, the scope of the actionable tools, co-production and types of evidence used. There were no significant differences in interpretations of the data.

### The scope and breadth of impact case studies linked to CLAHRCs

In total, we identified fifty-three impact case studies linked to a CLAHRC. There was an over two-fold increase from seventeen in REF 2014 to thirty-six in REF 2021. These fifty-three case studies form the basis of this analysis. All case studies are available in the public domain [13, 14].

In the REF 2014 dataset, searching identified fourteen potential impact case studies. Other approaches were used to ensure that all relevant impact case studies were located. This included reviewing published National Institute for Health and Care Research impact documents and direct contact with CLAHRCs to verify and identify any other impact case studies. This process identified three additional case studies and excluded one case study from the initial keyword search.

In the REF 2021 dataset the search terms produced thirty-seven results. Following an initial reading of these, it was agreed to exclude the *‘Enhancing research use, influence and impact in policy and practice’* which was submitted by the University of St Andrews because the focus of the case study was upon describing the influence of the research upon the strategies and practices of research funding bodies [28]. The National Institute for Health and Care Research investment into the CLAHRCs between 2013 and 2018 is described as one of these examples. Whilst the establishment of the CLAHRCs is pivotal, for the purposes of this study we wished to identify and illustrate the contribution of CLAHRCs which it was not possible to extract from this impact case study.

The identification process highlighted some challenges in the visibility of relevant CLAHRCs as there were gaps in those case studies which did not mention a CLAHRC. Even where there was reference to a CLAHRC there were some gaps in mentioning which specific CLAHRC linked to a particular case study. These impact case studies were verified through direct contact with the relevant CLAHRC and/or through investigation of the data sources in the impact case study.

Tables 2 and 3 provide an overview of the fifty-three impact case studies in REF 2014 and REF 2021 linked to CLAHRCs. The fifty-three impact case studies identified highlight the extensive links to CLAHRCs spanning the initial pilot phase to the newly established Applied Research Collaborations.

**Table 2:** List of identified impact case studies linked to CLAHRCs REF 2014: 17 in total

NIHR CLAHRC	Case Study Title	Unit of Assessment	Higher Education Institution	URL
Birmingham and Black Country	The benefits of early detection and intervention in psychosis	Psychology, Psychiatry and Neuroscience	University of Birmingham	http://impact.ref.ac.uk/CaseStudies/CaseStudy.aspx?Id=38809
Birmingham and Black Country	Epidural analgesia: Reducing instrumental delivery and side-effects of epidural analgesia during childbirth	Public Health, Health Services and Primary Care	University of Birmingham	http://impact.ref.ac.uk/CaseStudies/CaseStudy.aspx?Id=38799
Birmingham and Black Country	Hypertension: Improving routine diagnosis of hypertension in primary care	Public Health, Health Services and Primary Care	University of Birmingham	http://impact.ref.ac.uk/CaseStudies/CaseStudy.aspx?Id=38796
East of England	From research into mental capacity to clinical practice via Parliamentary statute: informing and implementing the Mental Capacity Act 2005	Psychology, Psychiatry and Neuroscience	University of Cambridge	http://impact.ref.ac.uk/CaseStudies/CaseStudy.aspx?Id=19852
Greater Manchester	Improving health through an evidence-based implementation programme	Business and Management Studies	University of Manchester	http://impact.ref.ac.uk/CaseStudies/CaseStudy.aspx?Id=28046
Greater Manchester	Mobilising knowledge to improve vascular health in the population of Greater Manchester	Business and Management Studies	University of Manchester	http://impact.ref.ac.uk/CaseStudies/CaseStudy.aspx?Id=28095
Leicester Northamptonshire and Rutland	Self-management in the prevention and treatment of type 2 diabetes: revolutionising patient care within usual healthcare practice	Clinical Medicine	University of Leicester	http://impact.ref.ac.uk/CaseStudies/CaseStudy.aspx?Id=35205
Leicester Northamptonshire and Rutland	Pre-diabetes and Type 2 diabetes: Risk-assessment tools for early detection and prevention	Public Health, Health Services and Primary Care	University of Leicester	http://impact.ref.ac.uk/CaseStudies/CaseStudy.aspx?Id=35209
Nottingham Derbyshire and Lincolnshire	Using interaction technologies to help people tackle the effects of stroke and other impairments.	Computer Science and Informatics	Nottingham Trent University	http://impact.ref.ac.uk/CaseStudies/CaseStudy.aspx?Id=12530
Nottingham Derbyshire and Lincolnshire	Implementing evidence-based community stroke services	Allied Health Professions, Dentistry, Nursing and Pharmacy	The University of Nottingham	http://impact.ref.ac.uk/CaseStudies/CaseStudy.aspx?Id=27120
North West Coast CLAHRC	Normal Childbirth: Leading international debate, evidence and action	Allied Health Professions, Dentistry, Nursing and Pharmacy	University of Central Lancashire	http://impact.ref.ac.uk/CaseStudies/CaseStudy.aspx?Id=2766
Peninsula (PenCLAHRC)	Identifying and promoting a new trauma treatment which could save over 100,000 lives a year	Public Health, Health Services and Primary Care	London School of Hygiene & Tropical Medicine	http://impact.ref.ac.uk/CaseStudies/CaseStudy.aspx?Id=41458
Peninsula (PenCLAHRC)	A new use for an old drug: administration of tranexamic acid to prevent trauma deaths from bleeding	Clinical Medicine	University of Leicester	http://impact.ref.ac.uk/CaseStudies/CaseStudy.aspx?Id=34476
Peninsula (PenCLAHRC)	Preventing suicides in non-clinical populations and settings	Public Health, Health Services and Primary Care	University of Exeter	http://impact.ref.ac.uk/CaseStudies/CaseStudy.aspx?Id=35583
South Yorkshire	Keeping Warm in Later Life (KWILLT)	Allied Health Professions, Dentistry, Nursing and Pharmacy	Sheffield Hallam University	http://impact.ref.ac.uk/CaseStudies/CaseStudy.aspx?Id=4948
South Yorkshire	Organisation of Maternity Care: A Cochrane systematic review on the midwife-led versus other models of care for childbearing women	Allied Health Professions, Dentistry, Nursing and Pharmacy	Sheffield Hallam University	http://impact.ref.ac.uk/CaseStudies/CaseStudy.aspx?Id=4949
South Yorkshire	Enhancing the use, influence and impact of research in policy and practice	Business and Management Studies	University of St Andrews	http://impact.ref.ac.uk/CaseStudies/CaseStudy.aspx?Id=35291

**Table 3:** List of identified impact case studies linked to CLAHRCs REF 2021: 36 in total

NIHR CLAHRC	Case Study Title	Unit of Assessment	Higher Education Institution	URL
CLAHRC East Midlands	Reduced falls and improved physical, mental and social health of older adults - implementation and adoption of the Falls Management Exercise (FaME) programme	Allied Health Professions, Dentistry, Nursing and Pharmacy	Glasgow Caledonian University	https://results2021.ref.ac.uk/impact/fb530e35-7447-4169-b735-184e65d1dd0f?page=1
CLAHRC East Midlands	Improving outcomes for older people with frailty and urgent care needs	Public Health, Health Services and Primary Care	The University of Leicester	https://results2021.ref.ac.uk/impact/30ff5c80-e0d2-44ba-9874-79d8a8a56f03?page=1
CLAHRC East Midlands	Increasing Physical Activity and Promoting Healthy Lifestyles to Prevent and Manage Diabetes	Sport and Exercise Sciences, Leisure and Tourism	The University of Leicester	https://results2021.ref.ac.uk/impact/6c9b745d-2d57-4b4e-957f-cbf4cada988f?page=1
CLAHRC East Midlands	Development and Implementation of Pre-Hospital Outcome Measures	Allied Health Professions, Dentistry, Nursing and Pharmacy	University of Lincoln	https://results2021.ref.ac.uk/impact/8cbb424a-26fe-4785-951e-393eaa22e36e?page=1
CLAHRC East Midlands	Transforming diagnostic assessment of attention deficit hyperactivity disorder (ADHD) in children and young people	Psychology, Psychiatry and Neuroscience	The University of Nottingham	https://results2021.ref.ac.uk/impact/b0a18b18-89c5-4c2c-b1da-360e96913add?page=1
CLAHRC East Midlands	Falls prevention amongst older people: Increased reach and further impact of interventions, uptake and adherence	Allied Health Professions, Dentistry, Nursing and Pharmacy	The University of Manchester	https://results2021.ref.ac.uk/impact/be3d165d-5c1b-4bc4-9d84-6c7d20b1146a?page=1
CLAHRC East Midlands; ARC East Midlands	Preventing, reducing and managing falls in adults	Allied Health Professions, Dentistry, Nursing and Pharmacy	The University of Nottingham	https://results2021.ref.ac.uk/impact/652a820f-29e7-4078-8124-f58b8b4af33e?page=1
CLAHRC East of England	Developing compassion and compassionate resilience in health care workers and family carers	Allied Health Professions, Dentistry, Nursing and Pharmacy	Anglia Ruskin University Higher Education Corporation	https://results2021.ref.ac.uk/impact/3e50056e-4f9b-4932-99cd-5a4c330afcc9?page=1
CLAHRC East of England Cambridgeshire and Peterborough; CLAHRC East of England; ARC East of England	PsyMaptic: a population prediction tool used by all NHS commissioners to design and provide early intervention services for people with psychotic illnesses according to population need	Psychology, Psychiatry and Neuroscience	University of Cambridge	https://results2021.ref.ac.uk/impact/ab9a35f6-a805-4caa-a467-96532815361b?page=1
CLAHRC East of England; ARC East of England	Enhancing health in care homes and communities	Allied Health Professions, Dentistry, Nursing and Pharmacy	University of Hertfordshire	https://results2021.ref.ac.uk/impact/654341c0-9c2d-4354-8b54-d5c5af946265?page=1
CLAHRC Greater Manchester	Evaluating and improving extended access to primary care	Business and Management Studies	The University of Manchester	https://results2021.ref.ac.uk/impact/b3800cc9-01b0-4c0a-a3a5-f8157c85e533?page=1
CLAHRC Greater Manchester	Optimising outcomes for people with venous leg ulcers	Allied Health Professions, Dentistry, Nursing and Pharmacy	The University of Manchester	https://results2021.ref.ac.uk/impact/1d278b74-0a27-4223-a396-f84cf0b26ee5?page=1
CLAHRC Greater Manchester; ARC Greater Manchester	Improving health service support for family carers during end-of-life care: implementing a Carer Support Needs Assessment Tool Intervention (CSNAT-I) and principles for organisation change	Allied Health Professions, Dentistry, Nursing and Pharmacy	The University of Manchester	https://results2021.ref.ac.uk/impact/7e6b6d0d-ac10-45bd-8cb5-37b533c35a1a?page=1
CLAHRC North Thames	Supporting patients’ medicines adherence using the Necessity Concerns Framework (NCF)	Allied Health Professions, Dentistry, Nursing and Pharmacy	University College London	https://results2021.ref.ac.uk/impact/af62ed4a-ed18-47ae-ac5d-c906e0969d0f?page=1
CLAHRC North Thames	Young Commissioner Framework: public and patient involvement in health and social care	Allied Health Professions, Dentistry, Nursing and Pharmacy	University of East London	https://results2021.ref.ac.uk/impact/66df317b-2b14-438f-a518-79fdbb495b7f?page=1
CLAHRC North West Coast	Mobilising knowledge from lived experiences to reduce social and health inequalities	Allied Health Professions, Dentistry, Nursing and Pharmacy	The University of Lancaster	https://results2021.ref.ac.uk/impact/588e5114-a790-4bb3-ab62-8fe2a820a4fd?page=1
CLAHRC North West Coast	Transforming the assessment and management of stroke survivors’ psychological and emotional needs to improve recovery	Allied Health Professions, Dentistry, Nursing and Pharmacy	University of Central Lancashire	https://results2021.ref.ac.uk/impact/1790c60c-82c2-4f6e-bd49-5e299ee67446?page=1
CLAHRC Northwest London	I-Hydrate: improving hydration in older people in health and social care settings	Allied Health Professions, Dentistry, Nursing and Pharmacy	The University of West London	https://results2021.ref.ac.uk/impact/7fcb28f7-ae34-46e6-a467-5793f24047cc?page=1
CLAHRC Northwest London	Improving patient care with seven-day services and better working practices	Public Health, Health Services and Primary Care	Imperial College of Science, Technology and Medicine	https://results2021.ref.ac.uk/impact/16ea09d5-6822-473e-8853-fca2cbd3ce29?page=1
CLAHRC Peninsula (PenCLAHRC)	Tranexamic acid treatment for patients with major trauma	Clinical Medicine	The University of Leicester	https://results2021.ref.ac.uk/impact/6d63ad2c-6de0-4797-b41c-269a9b9a362e?page=1
CLAHRC Peninsula (PenCLAHRC)	Co-creation of national policy and practical resources for preventing deaths by suicide	Public Health, Health Services and Primary Care	University of Exeter	https://results2021.ref.ac.uk/impact/dc42e842-c0d5-4b8c-af9d-d25980eac8e1?page=1
CLAHRC South Yorkshire	Changing national policy to expand the newborn bloodspot screening programme and to deliver economic benefits	Public Health, Health Services and Primary Care	The University of Sheffield	https://results2021.ref.ac.uk/impact/944cfeec-d234-4a77-a7e7-cb8be414bad4?page=1
CLAHRC Wessex	LifeGuide – Developing Internet-based Support for Healthcare	Psychology, Psychiatry and Neuroscience	University of Southampton	https://results2021.ref.ac.uk/impact/1bdac5c0-30f0-43c4-af58-eff901676595?page=1
CLAHRC West	Implementation science has rapidly scaled best practice to reduce preterm brain injury	Psychology, Psychiatry and Neuroscience	University of Bristol	https://results2021.ref.ac.uk/impact/43b92193-950e-4406-9445-6e1f59b613de?page=1
CLAHRC West	Prostate Cancer Screening trial informs guidelines, reduces harms to men from overdetection and avoids unnecessary health service costs internationally	Public Health, Health Services and Primary Care	University of Bristol	https://results2021.ref.ac.uk/impact/1031ccd1-935d-4814-bb97-ea0996bdf129?page=1
CLAHRC West Midlands	Enhancing clinical guidelines and professional training to transform the management of early psychosis and schizophrenia	Psychology, Psychiatry and Neuroscience	The University of Birmingham	https://results2021.ref.ac.uk/impact/ca847930-6a4d-49b6-9196-bbac31d5c212?page=1
CLAHRC West Midlands	Innovations in the NHS Bowel Cancer Screening Programme: Introduction of the Faecal Immunochemical Test (FIT)	Allied Health Professions, Dentistry, Nursing and Pharmacy	The University of Surrey	https://results2021.ref.ac.uk/impact/121aa346-fad3-4c48-8029-4f94de3486d0?page=1
CLAHRC West Midlands	Scaling Up Evidence-Based Healthcare Innovation in the West Midlands, Australia and India	Business and Management Studies	The University of Warwick	https://results2021.ref.ac.uk/impact/a0ce1781-f701-4915-be75-2a57667ed6a4?page=1
CLAHRC West Midlands	Youth Mental Health Policy and Service Reform: Regional, National and International Impact	Public Health, Health Services and Primary Care	The University of Warwick	https://results2021.ref.ac.uk/impact/5198d88d-ae2e-4eef-bb98-7b4c77576c13?page=1
CLAHRC Yorkshire and Humber	Improving patient safety using a behaviour change intervention	Psychology, Psychiatry and Neuroscience	The University of Leeds	https://results2021.ref.ac.uk/impact/51cd5de7-b8a1-4aa6-9d60-539b070782ce?page=1
CLAHRC Yorkshire and Humber	Improving service quality, uptake and health outcomes for patients with heart disease attending cardiac rehabilitation	Public Health, Health Services and Primary Care	University of York	https://results2021.ref.ac.uk/impact/5de32843-29af-4d30-8744-3466afa30641?page=1
CLAHRC Yorkshire and Humber	Enabling Healthcare Practitioners to Prevent Relapse and Support Self-management for People with Depression and Anxiety	Allied Health Professions, Dentistry, Nursing and Pharmacy	The University of Huddersfield	https://results2021.ref.ac.uk/impact/f68aef42-604f-4bd7-85cb-694dd6616686?page=1
CLAHRC Yorkshire and Humber; ARC Yorkshire and Humber; CLAHRC West Midlands	Development and national implementation of the electronic frailty index (eFI): impact on policy, health and care service delivery, and wider society	Public Health, Health Services and Primary Care	The University of Leeds	https://results2021.ref.ac.uk/impact/26618652-c2ff-4f0c-b821-12ad8876a999?page=1
ARC South West Peninsula	Person Centred Coordinated Care: Reforming National Health Policy and Accelerating National and International Care Delivery	Psychology, Psychiatry and Neuroscience	University of Plymouth	https://results2021.ref.ac.uk/impact/68749e9b-57a6-44bb-b890-48f0ab9b6fd5?page=1
ARC Yorkshire and Humber	Improving policy making via the Born in Bradford cohort	Psychology, Psychiatry and Neuroscience	The University of Leeds	https://results2021.ref.ac.uk/impact/d4219523-83bb-4a9d-97b5-8098455e5fe5?page=1
ARC North East and North Cumbria	An intervention that gives 529,000 people free and simple access to oral health guidance	Allied Health Professions, Dentistry, Nursing and Pharmacy	The University of Sunderland	https://results2021.ref.ac.uk/impact/01da03e2-5b30-46ed-b97c-b764c5fbccfc?page=1

**Fig. 1:**
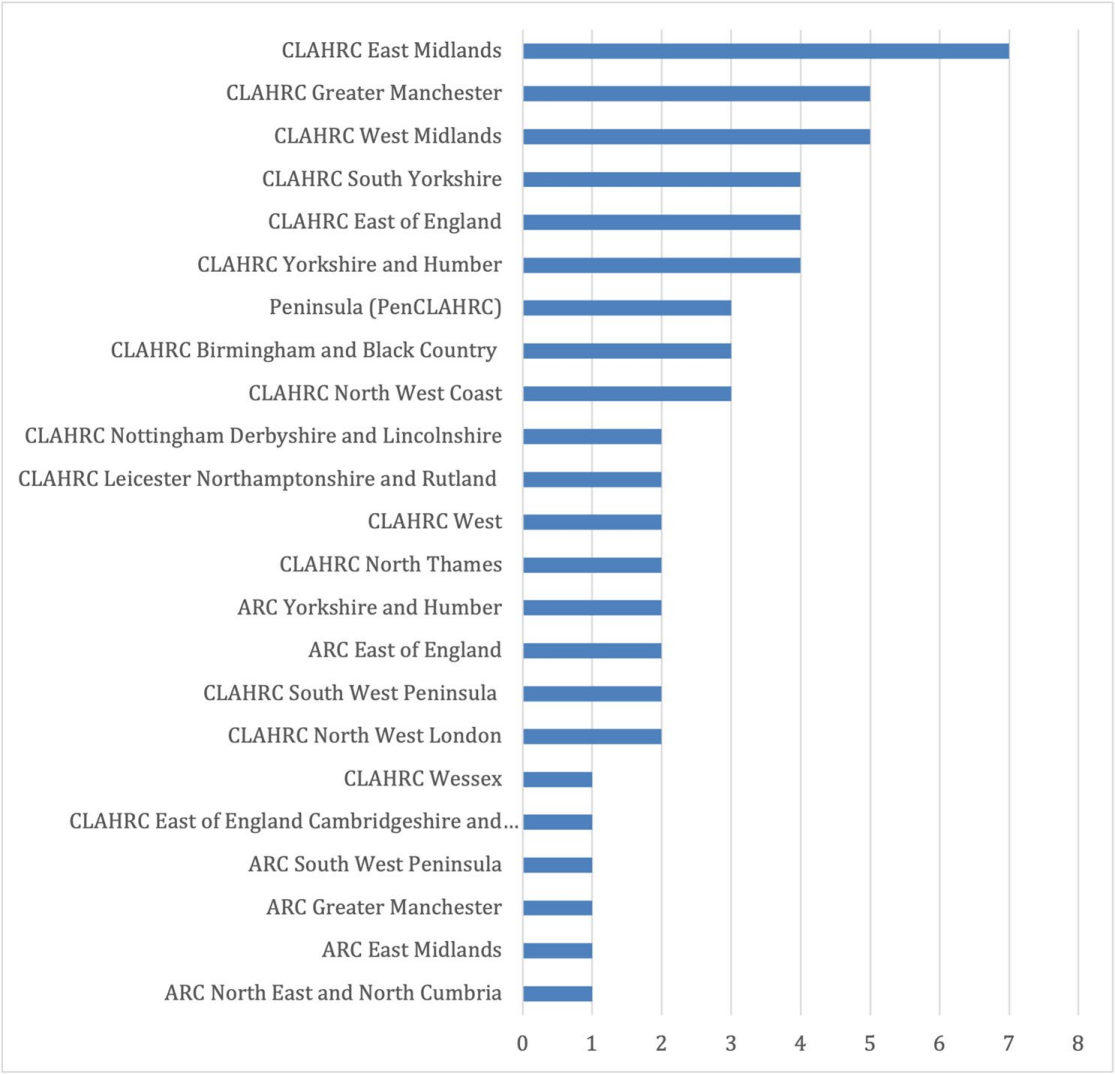
Overview by CLAHRCs linked to impact case studies in REF 2014 and REF 2021

Related CLAHRCs as anticipated were from the initial pilot of nine CLAHRCs between 2008 - 2013 in the REF 2014 dataset and the thirteen CLAHRCs between 2014 - 2019 in the REF 2021 dataset, with seven out of nine CLAHRCs represented in the pilot phase period and eleven out of thirteen CLAHRCs from the period between 2014 - 2019. Six out of fifteen of the newly established Applied Research Collaborations are also referred to in the REF 2021 dataset illustrating some of the on-going interactions and developments to research programmes. Longer term involvement of CLAHRCs from the pilot phase is also represented in at least three of the impact case studies submitted to REF 2021. Cross-CLAHRC collaboration is illustrated in two of the impact case studies. Fig. 1 provides a breakdown of the number of case studies linked to in REF 2014 and REF 2021 for each CLAHRC.

#### An overview of the higher education institutions

**Fig. 2:**
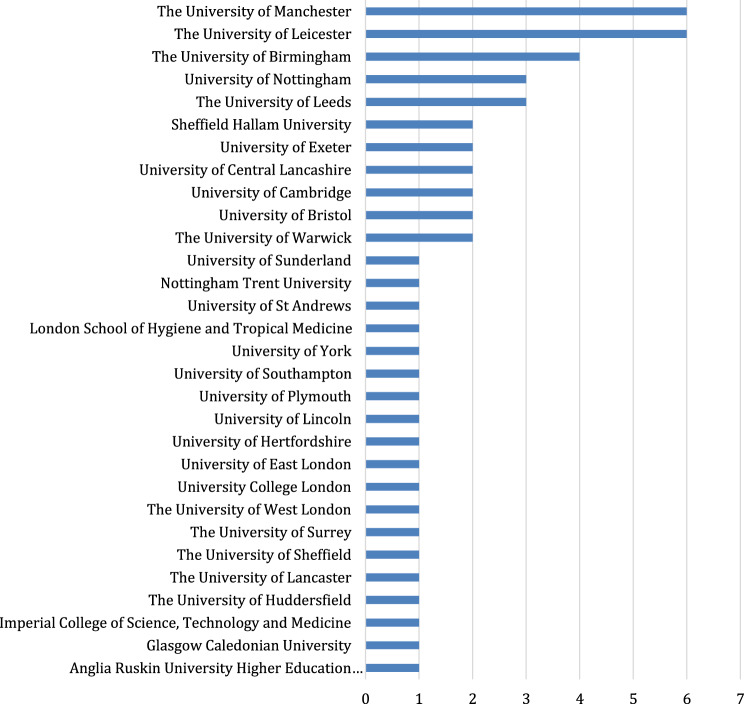
Breakdown by Higher Education institutions in REF 2014 and REF 2021

There were thirty universities in total who submitted impact case studies linked to a CLAHRC. The universities of Manchester, Leicester, Birmingham, Nottingham and Leeds all submitted three or more impact case studies. The universities of Sheffield Hallam, Exeter, Central Lancashire, Cambridge, Bristol and Warwick all submitted two impact case studies. Nineteen other universities all submitted one impact case study linked to a CLAHRC. Fig. 2 provides a breakdown of the higher education institutions involved.

In the UK, there are different types of universities reflecting their different origins and development. We categorised the types of universities into pre-1992, post-1992 and Russell Group. Post-1992 universities refers to universities which were granted university status following the Further and Higher Education Act of 1992 and are associated with the former polytechnics or other institutions. In contrast the pre-1992 universities refers to older universities, most of which were established through a royal charter. The Russell Group represents twenty-four universities and are associated with research-intensive activities.

In total, 74% of the case studies linked to a CLAHRC were from the older and/or Russell Group research-intensive universities.

#### Overview of which parts of the REF the case studies were submitted

The impact case studies were submitted predominantly to Panel A (medicine, health and life sciences) forming 87%. In addition, there were case studies submitted to Panel B (physical sciences, engineering and mathematics), (2%) and Panel C (social sciences) (11%). There were no case studies within Panel D (arts and humanities). Within Panel A the units of assessment which focus upon the allied health professions, dentistry, nursing and pharmacy and public health, health services and primary care form 62% of the identified case studies. Table 4 illustrates the breakdown of case studies by units of assessment in REF 2014 and REF 2021.

**Table 4:** Breakdown of case studies submitted to Units of Assessments in REF 2014 and REF 2021

Panel	Unit of Assessment (subject area)	Number of impact case studies submitted
Medicine, Health and Life Sciences	Allied Health Professions, Dentistry, Nursing and Pharmacy	20
Medicine, Health and Life Sciences	Public Health, Health Services and Primary Care	13
Medicine, Health and Life Sciences	Psychology, Psychiatry and Neuroscience	10
Social Sciences	Business and Management Studies	5
Medicine, Health and Life Sciences	Clinical Medicine	3
Physical Sciences, Engineering and Mathematics	Computer Science and Informatics	1
Social Sciences	Sport and Exercise Sciences, Leisure and Tourism	1

The REF dataset assigns up to three subject categories to each impact case study. Predominantly the subject areas are assigned within the medical and health sciences with some representation from the social sciences.

#### Information about the funders involved

In REF 2014 institutions were not required to mention the source of funding as part of the impact case study submission. We found that for REF 2014 all the impact case studies provided some information about funders. However where information was provided this was sometimes partial, for example in some cases information about the funder was provided but not the amount. In other cases some of the information about the funders was provided but this may not have encompassed all aspects of the research and pathway to impact.

The requirements changed for REF 2021 and institutions were asked to provide details of funders as part of the contextual information. These changes meant that for all case studies the funding information is generally more detailed than in REF 2014. Funding information is provided in most cases both within the case study and as part of the additional contextual information on the REF 2021 database. For analysis purposes, we drew upon the information in the case study and referred to the REF 2021 database to identify any further details. We found that some of the information about funders remained partial.

Whilst it is difficult to gather a standardised picture of the funders involved, we were able to gather information about the range of funders and the types of funders. Over eighty different funders were noted, with eight unknowns. There are a diverse range of funders involved with the majority of these being health research funding bodies. National Institute for Health and Care Research and National Institute for Health and Care Research CLAHRC/Applied Research Collaboration funding is dominant comprising 49%, followed by charitable/not for profit funders 12% and UK research councils 10%. Other funders include the National Health Service (NHS), UK government departments, industry and international organisations. Many case studies drew upon a series of awards. The size of an award can vary, for example a collaborative international randomised controlled trial which can attract significant funding and smaller awards to support specific aspects of the pathway to impact (for example supporting dissemination materials).

#### Impact categories and extent of the impacts

The REF dataset assigns a single impact category to each impact case study. It is an indicative guide to aid searching and as noted on the databases most impact case studies would encompass more than one type of impact [13, 14]. The eight summary impact types used in REF 2014 and REF 2021 are: political, economic, societal, technological, legal, environmental, cultural and health. The CLAHRC related impact case studies spanned three of the impact categories with health being the most dominant forming 74% followed by societal which formed 24%. Legal was also represented in one of the case studies. The case studies highlight that the extent of the impact goes beyond local or regional to national and international influence. International influence is found in 72% of the case studies with national forming 26%.

#### Indicative timescales between research and impact

We explored ascertaining the indicative timescales between the start of the research and impact commencing. We recorded the dates provided in the impact case study for the research and impact periods and reviewed the impact section of the case study. We excluded two case studies which were continuing in the REF 2021 dataset as the research and impact for these was continued from the REF 2014 period. The time periods between research and impact vary in the case studies with 29% of case studies showing eleven years and above. However, for 25% of case studies the time is between two to four years. This is followed by 18% of case studies showing between five to seven years and similarly 18% up to one year. The average timescale is just over ten years.

#### Types of evidence used

Impact case studies were required to provide evidence to corroborate their impact claims. Up to ten sources could be included. The types of evidence sources were categorised using the breakdown provided in the Higher Education Funding Council for England commissioned report on collecting research impact evidence [29]. In addition, we included an ‘other’ category to record evidence which did not fit neatly into the existing categories, such as toolkits produced. A variety of evidence sources are used including reports, media references, articles and testimonials. Predominantly, sources include references to published reports forming 49% of the evidence used, with testimonials being used in 22% of the case studies. Reports included references to guidelines, quality standards, evaluation reports, project reports, annual reports, government publications and national strategy documents. Testimonials ranged from individual users of research to representatives of organisations who used the research. Testimonials were not usually available publicly, though in some cases quotes had been included in the case study directly.

### Involvement of CLAHRCs in the impact case studies

We sought to identify the role that CLAHRCs have undertaken in the impact case studies. In 68% of the impact case studies we found that there was an explicit reference to a specific CLAHRC. In 25% a CLAHRC was mentioned but not which one and these were confirmed either through data sources in the impact case study or via verification with the CLAHRC. Whilst there is a mention of a CLAHRC in all the REF 2021 impact case studies we found that in four (8%) of the REF 2014 impact case studies there was no mention of a CLAHRC.

For 79% CLAHRC involvement encompassed contributing to the impact. In 8% there was both a contribution to the impact and on-going support for the research. In 6% there was reference to currently supporting the research. On-going contribution of the CLAHRCs may be occurring in other case studies though it was not possible to discern this from the text provided. We were unable to identify how the CLAHRC was involved in 8% of the case studies.

The impact case studies are structured into five descriptive sections consisting of summary of the impact; a description of the underpinning research; references to the research; details of the impact and sources to corroborate the impact. We found that 49% of the case studies refer to a CLAHRC in one section. In 32% a CLAHRC is referred to in two of the sections. In 13% a CLAHRC is referred to more than twice, with 2% showing that a CLAHRC is mentioned in all five sections of the case study. There is no mention of a CLAHRC in 8% of the case studies. Predominantly CLAHRCs were mentioned in sections two and three which describe the underpinning research which has contributed to the impact followed by section four which provides details of the impact being claimed and then section five evidence sources to corroborate the impact.

It was not possible to extract data sufficiently about the period from which CLAHRC support was noted with 55% of case studies where there was no information provided. Dates where provided were from 2008 onwards referring to the dates of funding from CLAHRC and/or where the date of CLAHRC support is specifically mentioned.

#### Analysis of a sample of the impact case studies

For illustrative purposes we selected eight impact case studies to highlight the range of disciplinary involvement and the contribution of the CLAHRCs. Table 5 provides details about the eight impact case studies including contextual background information, an outline of the impact claimed, funders involved, any collaborations, any actionable tools, characteristics associated with the impact and the main role of the CLAHRC. The sample consists of six case studies from REF 2021, including one which is a continuing impact case study from REF 2014 and two from REF 2014. Four of these are from Panel A (medicine, health and life sciences) with one from Panel B (physical sciences, engineering and mathematics) and three from Panel C (social sciences). The units of assessment include ‘Clinical Medicine’, ‘Allied Health Professions, Dentistry, Nursing and Pharmacy’, ‘Public Health, Health Services and Primary Care’, ‘Psychology, Psychiatry and Neuroscience’, ‘Computer Science and Informatics’, ‘Business and Management Studies’, and ‘Sport and Exercise Sciences, Leisure and Tourism’ exemplifying the breadth of subject areas.

**Table 5:** An exploration of a sample of 8 impact case studies with extracts from the case studies

Impact case study	Context	Intervention	Key impact	Main actionable tools	Main characteristics associated with the impact
**REF 2014: Improving health through an evidence-based implementation programme** [30] **The University of Manchester** **Panel C:** Business and Management Studies **Funders:** Refers to NIHR CLAHRC Greater Manchester. Builds on a range of funded projects, Knowledge Transfer Partnerships mentioned. **Collaborations:** NHS, The Stroke Association **Related NIHR CLAHRC:** Greater Manchester **Main role of NIHR CLAHRC:** Funder, implementation. Involved from 2008 onwards.	There was an identified gap in implementation. The NHS National Stroke Strategy (2007) included a structured assessment of people six months after being discharged from hospital. Structured assessment was one of the 20 Quality Indicators in the strategy. It was unclear what the assessment consisted of or who should carry it out [30].	CLAHRC ‘developed and supported the implementation of a review tool to identify needs and signpost to relevant support: the GM-SAT.’ [30]. It is claimed that CLAHRC involvement and testing of the tool facilitated the tool being fit for purpose which enabled confidence in using the tool in practice.	Influenced practice – the flexible assessment tool (GM-SAT) is used by a range of providers across England, fulfilling national strategy. Improved care for patients – an assessment of needs is undertaken, followed up with support so that needs can be met. The Stroke Association have carried out approximately 4000 reviews across England (at the time of publication) [30].	GM-SAT flexible, simple, free to use evidence-based tool. The Stroke Association evaluation report. GM-CLAHRC report on GM-SAT. NIHR Annual Report 2010/11.	Applied an implementation into practice model to develop the tool drawing upon rigorous research evidence. Evaluation embedded within. Multi-professional team with designated roles. Responded to national strategy and the need for structured assessment to be carried out in practice. Simple, free to use tool. Acceptability to professionals, aligned with national clinical guidelines. Endorsement from key stakeholders - NHS Stroke Improvement Programme, National Clinical Lead. Flexible in use and could be tailored to reflect local care pathways. Targeted publications.
**REF 2014: Using interaction technologies to help people tackle the effects of stroke and other impairments** [31] **Nottingham Trent University** **Panel B**: Computer Science and Informatics **Funders:** European Union (EU), Economic and Social Research Council (ESRC), Engineering and Physical Sciences Research Council, NIHR, CLAHRC, National Lottery. **Collaborations:** Universities of Nottingham, Athens, University of Putra Malaysia (UPM) and Stuttgart; industry; regional authorities and schools. **Related NIHR CLAHRC:** Nottingham Derbyshire and Lincolnshire **Main role of NIHR CLAHRC:** Funder	The research describes barriers to the adoption of new technologies such as virtual environments, serious games, assistive technologies and robotics. A programme of research was undertaken to address the need for efficacy and accessibility in clinical and educational settings [31]. The case study focuses upon stroke and people with intellectual disabilities.	The research team ‘undertook user sensitive inclusive design of new technological interventions and their subsequent evaluation in conjunction with users and beneficiaries’ [31].	It is stated that the research has ‘changed practice in schools, improved the employment skills of people with disabilities, informed standards, helped sustain a social enterprise, and has influenced the way practitioners across the EU conduct their own vocational training’ [31].	Produced a series of project reports to the EU. Series of research presentations, for example for the ESRC seminar series and the Swedish National Adult Rehabilitation Conference. Produced research results in an open source freely available format. Produced training resources and vocational training.	A long-established research programme which secured a series of successful grants. Used user sensitive inclusive design methods. Collaborative and multidisciplinary team with international partners, industry and educational involvement. The team were committed to open source development and open access results. Active dissemination for example at academic/practitioner events and through the international network of special educational schools and other key stakeholders. Capacity building with curriculum content and vocational training.
**REF 2021: Changing national policy to expand the newborn bloodspot screening programme and to deliver economic benefits** [32] **The University of Sheffield** **Panel A**: Public Health, Health Services and Primary Care **Funders:** NIHR CLAHRC South Yorkshire **Collaborations:** Sheffield Children’s Hospital NHS Foundation Trust. The team worked with the support of screening laboratories in the UK. **Related NIHR CLAHRC:** CLAHRC South Yorkshire **Main role of NIHR CLAHRC:** Funder, undertook evaluative pilot of expanding the newborn screening programme.	The case study states that identifying rare but serious diseases at birth is crucial to early treatment and the ability to save lives. Previously there was limited research into the potential health economic impact of false positives during screening of inborn errors of the metabolism. The research showed that there is widespread parental support for extended screening and that the number of false positives is a relatively small issue [32].	Blood spot heel prick test. The team undertook an evaluative pilot to expand the newborn screening programme to include five further inborn errors of the metabolism. The evaluation included UK screening laboratories nationally and an economic evaluation to assess the cost-effectiveness of expanded screening.	Expanded the newborn screening programme in the UK from screening for five metabolic conditions to nine. **Policy change:** informed decision by UK National Screening Committee (NSC) to include four additional conditions to the newborn screening programme, 2014 [32]. **Practice change: c**hanged screening for all newborn babies − 700,000 annually in England and Wales. **Patient outcomes improved:** between 2014 and 2018, 112 screen positive babies benefited from early detection helping to prevent severe disability and save lives [32]. **Economic impact:** Estimated cost-savings to the NHS overall. **International influence:** Policy influence in Spain, Bangladesh.	Project report of pilot evaluation and economic evaluation for UK NSC, 2013. Developed package of multimedia information and resources to support parents and professionals. Report to UK NSC on Severe Combined Immune Deficiency Screening, 2017. Health Technology Agency report to the Spanish Ministry of Health, 2019. International webinar by the Global Taskforce on Newborn Screening, 2020.	Collaborative with the NHS with an initial evaluative pilot. The team were involved directly with policymakers with findings reported to the UK NSC, 2013. National policy cites research findings. Changes in policy were integrated into practice through implementation and national roll-out of the expansion. The expansion was incorporated into the existing heel prick test infrastructure facilitating roll-out. The research team produced a peer-reviewed publication to evidence data since the implementation, 2020. Public Health England reported on audit data of screening to evidence uptake. International influence through Bonham’s role as President of the International Society of Neonatal Screening.
**REF 2021: Increasing Physical Activity and Promoting Healthy Lifestyles to Prevent and Manage Diabetes** [33] **The University of Leicester** **Panel C**: Sport and Exercise Sciences, Leisure and Tourism **Funders:** NIHR CLAHRC, and two other NIHR grants. **Collaborations:** Ingeus UK, (a service design and delivery company). The research involved the NHS and participants. **Related NIHR CLAHRC:** East Midlands **Main role of NIHR CLAHRC:** Funder. Piloted intervention.	The case study states that physical activity is key in the prevention and management of type 2 diabetes (T2D). The NHS Health Checks Programme was rolled out in 2008 for adults aged 40–75 with diabetes prevention a key target. It offered those individuals at high-risk a lifestyle programme. However, at the time there was insufficient evidence about how diabetes prevention in primary care could be effectively and economically delivered at scale [33].	Developed physical activity diabetes prevention programmes: *Walking Away from Diabetes* and *Let’s Prevent Diabetes.* The research team carried out a programme of research which included randomized controlled trials, piloting, evaluation and cost-effectiveness data in the development of the interventions.	The *Let’s Prevent Diabetes* programme is delivered nationally through *‘Healthier You: the NHS Diabetes Prevention Programme’*. There have been over 140,000 referrals to date. Patient benefits include ‘significant weight loss, improved glycaemic control and reduced diabetes risk’ [33]. National policy influence includes the National Institute for Health and Care Excellence (NICE) guideline ‘Type 2 Diabetes: Prevention in People at High Risk’, 2017; and the service specification for the NHS *‘Healthier You’*. Internationally the research has informed clinical practice guidelines, for example, in Canada, the USA and India. The *Let’s Prevent* programme was delivered in Western Australia, 2018.	Public Health England report ‘Evidence Review of Diabetes Prevention Programmes’, 2015. NHS Diabetes Prevention Programme, Healthier You, 2016. NICE Guideline, ‘Type 2 Diabetes: Prevention in People at High Risk’, 2017 update to 2012. Northern Ireland Department of Public Health Annual Report, 2014. NHS England Impact Analysis of implementing NHS Diabetes Prevention Programme, 2016–2021. International: Diabetes Canada, 2018, Physical Activity and Diabetes guideline; American Diabetes Association’s Standards of Care in Diabetes guidance, 2019; Joint Research Society for the Study of Diabetes in India and Endocrine Society of India practice recommendations, 2020.	Collaborative: with the NHS, industry and policymakers. National driver: the roll-out of the NHS Health Checks Programme included diabetes prevention as a key component. Policy influence: includes the team being commissioned by Public Health England; invitation to provide expert guidance by the NHS. Incorporated into international guidelines (Canada, USA, India). Collaboration with industry led to tailoring the *Let’s Prevent* programme to meet the service specification. Implementation of *Let’s Prevent *in 2016, with national roll-out in 2017. Adopted by Northern Ireland Department for Public Health and adapted for use. Recognition: winning entry in the ‘Quality in Care’ award, 2018. Availability of impact data to demonstrate outcomes nationally and internationally.
**REF 2021: Tranexamic acid treatment for patients with major trauma** [34] **The University of Leicester** **Panel A**: Clinical Medicine **Funders:** National Institute for Health and Care Research **Collaborations:** London School of Hygiene and Tropical Medicine; Peninsula CLAHRC. The research involved an international network and the NHS. **Related NIHR CLAHRC:** Peninsula (PenCLAHRC) **Main role of NIHR CLAHRC:** Initial implementation led by PenCLAHRC in conjunction with the research team. Provided one of the evidence sources for the case study.	It is stated that ‘bleeding after trauma is a global healthcare issue leading to millions of deaths every year’ [34]. The research team demonstrated that the antifibrinolytic agent, tranexamic acid (a drug that prevents clots from breaking down) is effective as a treatment for traumatic bleeding. The trial showed that ‘one life was saved for every 67 patients treated’ [34]. However, data showed that it was being administered later than the timeframe to be most effective.	Use of tranexamic acid (TXA) for the treatment of bleeding after trauma.	The research led to global change in trauma management protocols, saving an estimated 140,000 lives per year worldwide [34]. Changes were incorporated into the NHS England Best Practice Tariff (BPT) for major trauma (enabling additional per patient funding to NHS Major Trauma Centres). Further revision to the NHS BPT criteria in 2020 to administer TXA within one hour of injury (when it is most effective, changed from three hours previously). Nationally, the number of patients treated with TXA increased from 1% in 2010 to 85% in 2016. Estimates from NHS England indicate that these changes created an additional 1600 survivors by 2018 [34]. Economic analysis showed a cost of $64 per life year gained in the UK [34].	NHS England Best Practice Tariff revised for 2019/20; NHS England press release about use of TXA; NICE guideline, NG39 ‘Major Trauma: assessment and initial management’, 2016. European Trauma Bleeding Guideline updated, 2016; South Africa guidelines, 2018; global civilian and military guidelines; and global military protocols; global trauma training course - Advanced Trauma Life Support, updated 2018. CLAHRC Implementation Program NIHR CLAHRC Peninsula Briefing Document. Trauma Audit and Research Network (TARN) website.	**Collaborative:** with an international network and the NHS. **Identified need:** demonstrated effectiveness of TXA in trauma, however it was being given later than the timeframe to be most effective. The drug was relatively cheap and already available. **Policy influence:** strong and on-going links with policymakers with research team involvement in the guidelines group. Incorporated into guidelines and protocols worldwide - civilian and military. The use of TXA was included in the NHS payment by results BPT. **Practice support:** produced an implementation briefing document. Incorporated into global trauma training course - Advanced Trauma Life Support, updated 2018. **Monitoring of implementation and outcomes:** audit data was administered by TARN. Coats a research team member chaired TARN.
**REF 2021: Scaling Up Evidence-Based Healthcare Innovation in the West Midlands, Australia and India** [35] **The University of Warwick** **Panel C**: Business and Management Studies **Funders:** University Hospitals Birmingham NHS Foundation Trust; Sandwell and West Birmingham Hospitals NHS Trust; The University Hospitals Coventry and Warwickshire NHS Trust; Heart of England NHS Foundation Trust; two NIHR grants; Economic and Social Research Council; Australian Research Council; Department of Health. **Collaborations:** University of Nottingham. Co-produced with practitioners. **Related NIHR CLAHRC:** CLAHRC West Midlands **Main role of NIHR CLAHRC:** Undertook evaluation of pilot CLAHRCs. Development of a new implementation model which was adopted by CLAHRC West Midlands. Provided one of the sources to corroborate impact.	The CLAHRCs were established by NIHR to translate research evidence into practice to improve healthcare. The Cooksey Review of 2006 highlighted the need to address gaps between research and practice [35]. The team undertook an evaluation of the pilot CLAHRCs, developed a new implementation model in conjunction with colleagues from The University of Nottingham and applied this to a range of healthcare settings.	Developed a new implementation model, founded on organisation science research to translate evidence from research into practice. The model ‘improved knowledge brokering, distributed leadership towards the middle of the organisation and strengthened human resource management’ [35].	The implementation model has been applied to a range of healthcare settings in the West Midlands. It is claimed that it has led to benefits in maternity, musculoskeletal disease, mental health and cancer services both for staff and patients. The model has also been adopted internationally to improve health service delivery. In India in the implementation and scale up of an early intervention service for young people with psychosis and in Australia with Academic Health Science Centres to improve implementation and scale up of evidence-based healthcare innovation.	Implementation model. Two articles: the design and implementation of an obstetric triage system and youth mental health in India.	Collaborative with the NHS. Currie as a research team member played a key role within CLAHRCs from the early set-up. Developed new implementation model. Adopted co-production approach. Adopted and integrated into the CLAHRC West Midlands and applied to a range of NHS services. Undertook international application of the model.
**REF 2021: PsyMaptic: a population prediction tool used by all NHS commissioners to design and provide early intervention services for people with psychotic illnesses according to population need** [36] **The University of Cambridge** **Panel A**: Psychology, Psychiatry and Neuroscience **Funders:** Stanley Medical Research Institute, St Bartholemew’s and the London Hospitals’ Trust, two NIHR grants, NIHR CLAHRC Cambridgeshire and Peterborough, NIHR CLAHRC East of England, NIHR ARC East of England, Medical Research Council, Department of Health, Sir Henry Wellcome. **Collaborations:** Queen Mary University London, King’s College London University. The research involved NHS services. **Related NIHR CLAHRC:** CLAHRC East of England Cambridgeshire and Peterborough; CLAHRC East of England; ARC East of England **Main role of NIHR CLAHRC:** Funder (since 2008 – current). States that making the PsyMaptic tool freely available was a process facilitated through working with the CLAHRC. Produced information about the use of the tool as a CLAHRC BITE which is cited as one of the sources to corroborate impact.	Conditions such as schizophrenia and other psychotic disorders have considerable personal implications. Early treatment is associated with better outcomes and with reducing the overall cost of care. ‘In England, specialist NHS early intervention services (EIS) were set up in the 2000s to address this but resources were not distributed across the country in a way that matched need’ [36].	Developed PsyMaptic: a free, population prediction tool to design and provide early intervention services for people with psychotic illnesses according to population need [36].	**Informed policy:** In 2014, PsyMaptic was used by Public Health England as part of their ‘Fingertips health profiles’, these are data tools designed to ‘support commissioning groups in reducing inequalities and improving health and wellbeing’ [36]. In 2016, national NICE guidance recommended that all Clinical Commissioning Groups (CCG) use PsyMaptic in recommissioning of EIS services. The 2020 NICE update continues to recommend the use of PsyMaptic. **Improved access to treatment for patients:** ‘Access to EIS in England within two weeks of a first psychotic episode has now increased from 33% of patients in 2014 to 76% in 2018/19, exceeding NHS targets of 50%’ [36].	PsyMaptic tool. Public Health England ‘Fingertips health profiles’, data tools. 2014. Guidelines produced by the Joint Commissioning Panel for Mental Health, 2015. NHS England guidance, 2015. Guidance from NHS England, the National Collaborating Centre for Mental Health and NICE: Implementing the Early Intervention in Psychosis Access and Waiting Time Standard: Guidance, 2016, 2020. Health Education England programme of training. CLAHRC East of England BITE. Annual Report of the Chief Medical Officer 2013 (published 2014).	Sustained programme of funding and on-going, including a Department of Health commissioned study with CLAHRC support since 2008. Developed PsyMaptic as a freely available, open-source tool. Incorporated by Public Health England into the ‘Fingertips health profiles’, data tools. Incorporated into national guidelines with guidance provided for implementation. Research team members were invited onto the NHS England Expert Reference Group as part of the planning process. Prof Jones appointed clinical lead for implementation of the standard by NHS England Midlands and East. NHS England provided investment to support workforce development with a Health Education England training programme. NHS England commissioned a survey - providing evidence of use of the tool by all CCGs. Evidence of patient benefit provided through routine monitoring of national data - Royal College of Psychiatrists’ National Clinical Audit of Psychosis.
**REF 2021: Co-creation of national policy and practical resources for preventing deaths by suicide** [37] **The University of Exeter** **This is a continuing impact case study from REF 2014** **Panel A**: Public Health, Health Services and Primary Care **Funders:** Public Health England (PHE); NIHR CLAHRC; Medical Research Council. **Collaborations:** The research involved working with, the Samaritans, Network Rail Ltd, the National Trust, the Department for Transport, East Sussex County Council, Middlesex University, the Centre for Crisis Psychology, Jonny Benjamin MBE (Channel 4 film maker), The Alliance of Suicide Prevention Charities (TASC) and a panel of people bereaved by suicide. **Related NIHR CLAHRC:** South West Peninsula CLAHRC (PenCLAHRC) **Main role of NIHR CLAHRC:** Funder	It is stated that over 6000 people die by suicide in the UK. For young people suicide is the leading cause of death and it has higher rates among those from deprived backgrounds [37]. There had been limited scope for clinical intervention because many people who die by suicide have not had recent contact with services [37]. The research team focused on public health community-based approaches for the prevention of suicide in public places and developing understanding of what lay people can do and what skills or resources they need.	Developed guidance to inform the management of high-risk suicide locations. Developed a training module based upon the *Stranger on the Bridge* study. With the TASC and a panel of people bereaved by suicide produced a public education leaflet, *‘It’s safe to talk about suicide’.*	**Informed national public health policy and changes to practice** ‘Enabled all of England’s 152 public health teams to deliver the national suicide prevention strategy, with 78% including specific actions to reduce suicides in public places’ [37]. Informed NICE guidelines, 2018 on preventing suicide in community and custodial settings and informed Highways England’s strategy and training [37]. **Contributed to national public health campaigns for the prevention of suicide in the UK** The *‘It’s safe to talk about suicide’* leaflet ‘has been used by at least nine local authorities, five universities, the Royal National Lifeboat Institution and the Fire Officers Association and directly shaped national public health campaigns empowering lay people to play an active part in suicide prevention’ [37].	National guidance on preventing suicides in public places. NICE Guideline on ‘Preventing suicide in community and custodial settings’, 2018. All Party Parliamentary Group (APPG) report on ‘Suicide and Self-Harm Prevention’, 2015. Highways England strategy and guidance for preventing suicides on the strategic road network. Guidance for developers and the construction industry. Training module for traffic officers. ‘Local suicide prevention: a practical resource’ guidance, 2016. Review of local suicide prevention plans report. Local Authority sector-led improvement programme – included national masterclasses/webinars and a Must-Know Guide. Public education leaflet, *‘It’s safe to talk about suicide’* - hard copy and online. BiggerIssues campaign by the Campaign Against Living Miserably and the TalkThroughTheTaboo campaign.	Collaborative - working with partners from public health, the voluntary and community sector, industry and people affected by suicide. The team won a contract from PHE to revise the guidance for local authority public health teams. Incorporated into national guidance which was circulated to all local authorities with a letter from the Secretary of Health. This led to the development of site-specific plans. Invited to act as an advisor for a range of organisations (for example, public health bodies, local authorities, construction industry, the National Trust). Cited by the APPG on Suicide and Self-Harm Prevention, 2015. The APPG report led to PHE to commission local authority practical resources with Owens invited to contribute to its development. The Local Authority sector-led improvement programme was launched to provide support at a local level. Produced a public education leaflet. The leaflet enabled bespoke adaptation through a Creative Commons license and was available as a hard copy and online. It was translated for use in Malaysia. Developed a training module to support practitioners for Highways England. Active public education campaigns involving key organisations and partners.

The eight case studies cover research which addresses health and care needs including implementation of a stroke assessment review tool, the use of interaction technologies to help people with stroke and people with disabilities, the newborn bloodspot screening programme, the prevention and management of diabetes, trauma treatment and management, the application of an implementation model in healthcare settings, early intervention services for people with psychotic illnesses and guidance for suicide prevention. The sample includes research which spans local, national and international influence.

All the case studies involve collaborations, for example with other universities, international partners, with the National Health Service and practitioners, with charities, industry, regional authorities and people using services. A diverse range of funders is listed including the National Institute for Health and Care Research, CLAHRCs and Applied Research Collaborations, UK research councils, National Health Service Trusts, the National Lottery, the European Union, the Stanley Medical Research Institute (United States of America (USA)) and professional organisations such as the Royal College of Midwifery. The case studies highlight how since the pilot phase of the CLAHRCs there has been impact from research related programmes. Examples of CLAHRC involvement from the pilot phase of CLAHRCs (2008–2013) include the ‘*Improving health through an evidence-based implementation programme’* (University of Manchester) [30], the *‘Changing national policy to expand the newborn bloodspot screening programme and to deliver economic benefits’* (University of Sheffield) [32] and the *‘Scaling up evidence-based healthcare innovation in the West Midlands, Australia and India’* (University of Warwick) [35] and the continuing impact case study *‘Co-creation of national policy and practical resources for preventing deaths by suicide’* (University of Exeter) [37].

There are other case studies which draw upon established programmes of work with links to the pilot phase of the CLAHRCs such as the *‘Tranexamic acid treatment for patients with major trauma’* (University of Leicester) [34] and the ‘*Increasing physical activity and promoting healthy lifestyles to prevent and manage diabetes’* (University of Leicester) [33]. Within the REF 2014 submissions the ‘*PsyMaptic: a population prediction tool used by all NHS commissioners to design and provide early intervention services for people with psychotic illnesses according to population need’* (University of Cambridge) [36] shows sustained CLAHRC involvement from the pilot phase to on-going support from the National Institute for Health and Care Research Applied Research Collaboration.

Within the sample, details about the role and contributions of the CLAHRCs are varied. In the ‘*Improving health through an evidence-based implementation programme’* (University of Manchester) [30] and the *‘Scaling up evidence-based healthcare innovation in the West Midlands, Australia and India’* (University of Warwick) [35], CLAHRC involvement in the implementation models and leadership roles is embedded throughout. The examples show a range of CLAHRC contributions including identification of research priorities and needs, funding support, research design, co-production, implementation, involvement in piloting interventions, evaluation activities, publications related to the research, production of tailored resources for practitioners and people using services, dissemination activities, and providing evidence sources to corroborate impact claims. In some cases it was not possible to ascertain CLAHRC involvement beyond a description as a ‘funder’.

### Characteristics associated with the impact case studies

We explored characteristics associated with achieving impact for each of the case studies. Specific causal effects can be difficult to establish between research and impact, for example impact may not be a linear process, or there may be direct and indirect factors involved [38]. In addition, the format of the case study is focused upon reporting impacts rather than the processes involved in achieving impact. We were able to extrapolate enabling factors which align with other reported work on pathways to impact from research and include some additional areas, whilst recognising that impact is multi-faceted and not necessarily prescriptive [21, 22, 26, 38]. In the context of CLAHRC related case studies, these include:

- research which responds to an identified need or gap (sometimes commissioned research by national bodies);
- external ‘drivers’ for the research;
- collaborative work;
- multidisciplinary involvement;
- co-production;
- strong and on-going links with policymakers (sometimes with invitations to present/advise);
- involvement in guideline development;
- incorporation into policy/guidelines;
- support for implementation;
- development and delivery of training/educational materials for professionals and the public;
- the production of actionable tools to facilitate use in practice;
- piloting of interventions as test beds prior to scaling up;
- evaluation of interventions embedded as part of the research (with cost-effectiveness included where appropriate);
- targeted dissemination activities;
- translation of resources for use in other countries;
- making resources freely available to ensure access;
- flexibility built in to enable use in a variety of contexts;
- availability of sources of evidence to demonstrate changes (for example, routine audit data already collected nationally).

The case studies demonstrate the commitment of the research teams to optimising the impact of the research working in conjunction with other partners and using a range of routes and mechanisms within the context of each research programme and its wider environment.

## Discussion

Our review has identified fifty-three impact case studies linked to a CLAHRC in the REF 2014 and REF 2021 datasets. Locating relevant case studies linked to a CLAHRC relied on these being mentioned and/or verified for their inclusion within this analysis. Whilst efforts were made to ensure that all relevant case studies were included it is possible that we may still have missed some case studies, for example if the funder information was not fully completed in the submitted case study and/or if the CLAHRC was not aware of a case study being submitted. The changes to the submission requirements which led to information about funders being included have meant that for the REF 2021 dataset all the identified impact case studies specifically refer to a CLAHRC. This has improved the visibility of the CLAHRCs in the submissions. However, there is scope for the visibility of the CLAHRCs to be further improved. We found that in 25% of impact case studies whilst there was reference to a CLAHRC there was not a direct mention of which CLAHRC without further investigation.

The impact case studies linked to a CLAHRC were predominantly within Panel A (medicine, health and life sciences) reflecting a key focus of the CLAHRCs upon improving patient outcomes and services through partnership working with the National Health Service. There were signs of wider disciplinary contributions with 13% of the case studies within Panels B (physical sciences, engineering and mathematics) and C (social sciences) combined. The increase from seventeen impact case studies in REF 2014 to thirty-six in REF 2021 is encouraging and highlights how the evolution of the CLAHRCs can support the on-going generation of REF impact case studies.

There is also a need to exercise some caution about potentially over-representing the contribution of a CLAHRC in the REF impact case studies. The impact case studies were written from the perspective of the higher education institutions who wished to ensure the best possible assessment outcomes. Understandably the focus is upon the strengths of the submitting organisation and not upon showcasing the CLAHRCs or other partners who may be involved. In addition, the dates of CLAHRC involvement are mostly unclear with 55% of case studies where this is not noted. Further details about CLAHRC involvement (where possible within the submission requirements) would aid future analysis.

The analysis is based upon the impact case studies submitted to the REF and specifically drew upon the case studies available to search in the databases. There are some limitations to using the REF dataset for analysis purposes and these have been highlighted in published reports [17, 18, 39]. For example, the specific eligibility criteria and the purposes of the REF for assessment decisions means that the REF presents a highly selective view of impact. The REF case studies did not intend to, nor do they necessarily represent all impact that is occurring across the sector [17, 18, 39].

In addition, the impact case studies are described predominantly in positive terms focusing upon the benefits and changes that can be attributed to the research [17]. Universities reasonably selected those case studies which would potentially score higher against the REF criteria. Impact was assessed highly across the REF with 88% of impact case studies achieving a combined 4*/3* level in REF 2021, an increase from 84% in REF 2014 [15, 16]. Analyses of the impact case study dataset suggest that whilst the REF dataset may not have intended to provide a comprehensive picture of research impact it contains a rich resource of impact case studies showcasing diverse examples of impact across disciplinary areas. The REF dataset has been acknowledged as being sufficiently robust for analysis purposes with further examination of the impact case studies encouraged [17].

The REF quality assessment exercise provides a significant driver to demonstrating impacts from research with funding allocated to universities based on assessment decisions. However, there are other significant imperatives to demonstrating research impact. There can be a range of reasons why impact from research matters, for example demonstrating impact is relevant for funders in identifying return from investment and to provide evidence for future investment decisions; impact can form part of organisational reporting and annual reports; a track record of demonstrating impact and optimising impact can form part of future funding applications; in addition there are societal, individual and personal imperatives to achieving impact [10, 40, 41].

Alongside the REF impact case studies, organisations and funders have also been collating impact case studies. Examples include *‘The National Institute of Health and Care Research at 10 years: An impact synthesis of 100 impact case studies’* [20] and the National Institute of Health and Care Research CLAHRC report on *‘World Class Research Making a Difference’* (which brings together twenty-three impact case studies) [25] and the Medical Research Council Impact Showcase [42]. These complement the impact case studies in the REF datasets, with some of the examples reflecting case studies which were submitted to the REF. Taking account of this wider context of research, it is therefore imperative to continue to value and nurture a range of impacts including for REF submissions and other purposes.

Given that there are complexities in determining the timescales between research and impact it was not possible to be precise about these time periods [43]. This is an area which would benefit from further discussion with case study authors. It is also possible that the REF eligibility criteria for the impact period may have excluded describing any earlier impacts which may have arisen. The timescales provide some indication drawing upon the information within the case studies. They may help to shed some light upon how long it takes between research and impact, add to our understanding of what time points it is reasonable to consider and offer some insights into instances where rapid research evidence may be accelerated to inform policy or practice, for example societal needs in response to the COVID-19 pandemic [43, 44].

In examining the role and contribution of the CLAHRCs in the REF impact case studies there are a range of contributions which span health and care priorities at both a local and national level. The contributions of the CLAHRCs in the main encompass acting as a funder of the research, providing support for implementation into practice, research design, piloting of interventions and evaluation, support to extend the reach of the impact and contributions towards providing evidence to corroborate impact claims. In some cases the involvement of the CLAHRCs builds upon established research programmes, whilst some of the case studies also emerged directly with the CLAHRCs.

Greenhalgh and Fahy (2015) comment that in models such as CLAHRCs that research and impact are not necessarily separate and that these may be ‘two dimensions of co-produced activity’ [22]. It is possible that where CLAHRCs are identified as a ‘funder’ this may potentially underestimate the contribution of a CLAHRC. For example, as a funder CLAHRCs are potentially able to leverage and add value to the pathway to impact with contributions which may include identifying local/regional health and care research priorities, supporting implementation research, capacity building, access to clinical and other infrastructure, acting as methodological testbeds and developing participatory and coproduction design methods. Co-production and participatory research processes are characteristics associated with CLAHRCs and these are embedded explicitly in some of the identified case studies. The lack of visibility of co-production in the impact case studies has been highlighted [22] and this a strength of the CLAHRCs which could potentially be enhanced further for future case studies.

There is limited evidence of cross-CLAHRC working in the impact case studies with two out of fifty-three referring to the involvement of more than one CLAHRC. Overall the impact case studies which refer to CLAHRCs are focused upon the contribution of one CLAHRC and in some cases on-going contribution through the different phases of the same CLAHRC. It is possible that there may be cross-CLAHRC working in other impact case studies which has not been described. In addition, it is likely that the local and regional framework best supports developments with the local CLAHRC. Given that the CLAHRCs are a national network there may be scope for further cross-CLAHRC working to optimise impacts at a national and international level and to share practice. In addition, Kislov et al. (2018) suggest in their evaluation of CLAHRCs that there is scope to enhance capacity development across CLAHRCs [23].

Capacity development formed a core function of the CLAHRCs with skills development forming a key theme [45]. Research capacity development has been defined as ‘a funded, dynamic intervention operationalized through a range of foci and levels to augment ability to carry out research or achieve objectives in the field of research over the long-term, with aspects of social change as an ultimate outcome’ [46]. It is associated with increasing the ability of people and the organisations that they work for to develop skills, changes to professional training, curricula and methods. The CLAHRCs undertook a wide range of approaches to build the capacity of health and care professionals and managers including workshops and events to support continuing professional development, internship opportunities, and postgraduate research degree and research fellowship opportunities as well as providing access to the range of CLAHRC activity [45, 47–49].

The REF guidance includes contributing to continuing personal and professional development as an indicator of impact [12]. Within the CLAHRC related impact case studies there are examples of capacity development including professional development opportunities. This includes development and provision of training to support practitioners, such as the investment by the National Health Service England to support workforce development through a national Health Education England training programme as part of delivering key interventions supporting access to early intervention services [36], and the impact on education and training of practitioners as part of a national falls prevention programme to facilitate adoption into practice [50].

Within the impact case study format, it was not possible to ascertain the ways in which CLAHRC support may have been used to lead to other forms of improving professional development such as higher degree research. There is some limited reference to this within the impact case studies identified, for example the *‘Keeping Warm in Later Life’* (Sheffield Hallam University) impact case study refers to CLAHRC support co-funding one doctoral student and one professional doctorate student [51]. It is feasible that examples of these forms of support were collated as part of the annual reports produced by CLAHRCs as well as other published evaluations and reports [45, 49].

A wide and diverse range of impacts is described in the sample of case studies, including patient benefits, interventions to prevent health related issues, better support and care, policy and practice changes and international benefits of research programmes. The mechanisms and routes associated with the impact case studies are diverse thus complementing the findings of REF analyses which in the REF 2014 dataset highlighted as many as 3709 unique pathways to impact [17, 52]. Whilst direct causal effects are difficult to attribute our analysis highlights that collaborative and partnership working is pivotal to optimising impact and this is characterised through the work of teams.

Within the context of CLAHRC related impact case studies enabling factors include responding to identified priorities which may emerge from local or regional needs or from a policy driver, collaborative and partnership working, the creation of actionable tools to aid use into practice, the development and delivery of training packages, research designs which incorporate co-production and implementation into practice, piloting of interventions to facilitate use, establishing strong and ongoing links with policymakers, including as members of guideline development groups, the development of public resources (freely available), together with targeted and active dissemination strategies. These findings align with and add to other work which has examined research impact emphasising the multi-faceted and context-specific nature of optimising impact from research, collaborations and engagement activities [38].

Research impact assessment continues to evolve internationally as governments and funders seek to demonstrate the societal impact of their investments [6, 9]. The REF exemplifies one model for assessing societal impact as part of a national quality assessment system. Internationally, countries are developing and incorporating impact assessment at scale [9]. Some countries have adapted the UK REF impact case study format into their systems; for example, the Swedish Research Council Strategic Research Centres have been submitting impact case studies since 2019 and Hong Kong’s 2020 Research Assessment Exercise has incorporated impact case studies in assessment and produced a searchable database [9, 53, 54]. Other countries such as the Netherlands, Spain, Finland, Australia and the USA have been developing and taking account of evaluating research impact [9]. The global multidisciplinary community of practice for research impact assessment is growing with a set of principles and guidelines which have been published to help researchers and practitioners, drawing upon the experience from the International School on Research Impact Assessment [40, 55].

Research impact is set to continue and evolve as part of the international agenda. Our findings have shown that collaborative partnerships are an integral feature of achieving impact. Those collaborations can be at multiple levels including local, national and international. It remains central for governments and funders to continue to provide opportunities to facilitate research collaborations nationally and internationally. For researchers and practitioners this analysis shows the benefits and value of nurturing partnerships and undertaking collaborative research activities to optimise impact. As the impact case studies show there can be a blend of funders involved. The UK REF has evolved with information about funders required routinely as part of the 2021 submissions. In this analysis, information about the funders has helped to illuminate the range of funders involved and their contributions to the pathway to impact and where this is not already in place or partially completed this would enhance future submissions both nationally and internationally.

Decisions about the next UK REF 2029 have been announced with planning underway. Impact remains an integral component of the quality assessment exercise and as in REF 2021 will form 25% of the assessment [56]. Based on our findings and emerging plans for the next REF the current National Institute for Health and Care Research Applied Research Collaborations are potentially in a strong position to make a valuable and greater contribution to future REF submissions and to demonstrating impact for other purposes.

## Conclusions

Our findings illustrate that the partnership model exemplified through the CLAHRCs with a focus upon applied health research and implementation into practice has made a significant contribution to the REF impact case studies, showing an increase in volume within successive cycles of the REF exercise. There is scope to further enhance the visibility of the CLAHRCs/Applied Research Collaborations in future submissions, for example through providing details about which specific CLAHRC/Applied Research Collaboration is linked and to including information which can help to illuminate the role and involvement of the CLAHRC/Applied Research Collaborations where possible within the case study format. Capacity development including continuing professional development is an integral feature of facilitating research into practice and policy. Future REF and other impact case study submissions may wish to consider making more explicit the support and role that collaborative networks and funders such as the CLAHRCs have contributed to a range of professional and personal development opportunities. There were limited examples of cross-CLAHRC impact case studies, and it would be useful to consider cross-collaborative working in relation to optimising impact and sharing practice for future impact case studies. For governments, funders and practitioners, as the REF is a periodic and selective snapshot of impact it is imperative that impact continues to be valued and supported for a range of purposes and for the benefit of different users of research.

## Electronic supplementary material

Below is the link to the electronic supplementary material.

Supplementary Material 1

## Data Availability

This study uses data from the UK Research Excellence Framework (REF) 2014 and REF 2021 impact case studies. The REF dataset of impact case studies is available in the public domain for REF 2014 at http://impact.ref.ac.uk/CaseStudies/and for REF 2021 at https://results2021.ref.ac.uk/impact. The REF impact case studies are published under a Creative Commons BY 4.0 International License which enables sharing and use within the license.

## Declarations

### Transparency declaration

The manuscript is an honest, accurate, and transparent account of the study being reported; no important aspects of the study have been omitted; any discrepancies from the study as planned have been explained.

## Abbreviations

AIMS: Association for Improvements in the Maternity Services
APPG: All Party Parliamentary Group
ARC: Applied Research Collaboration
BAP: British Association for Psychopharmacology
CCG: Clinical Commissioning Group
CLAHRC: Collaboration for Leadership in Applied Health Research and Care
CPD: Continuing Professional Development
EIS: Early Intervention Services
EU: European Union
NCT: National Childbirth Trust
NHS: National Health Service
NICE: National Institute for Health and Care Excellence
NIHR: National Institute for Health and Care Research
NSC: National Screening Committee
PHE: Public Health England
RCM: Royal College of Midwives
REF: Research Excellence Framework
TARN: Trauma Audit and Research Network
TASC: The Alliance of Suicide Prevention Charities
UK: United Kingdom
USA: United States of America
