# Supplementary material for: An analysis of the contributions of the Collaborations for Leadership in Applied Health Research and Care to the impact of research in the UK Research Excellence Framework 2014 and 2021
Source: BMC Health Serv Res. 2026 Mar 26;26:630. doi: 10.1186/s12913-026-14048-6 (PMC13141381; doi:10.1186/s12913-026-14048-6)
Supplement: Supplementary file 1 — Supplementary Material 1 [file 12913_2026_14048_MOESM1_ESM.docx]

**Appendix 1: List of** **Collaborations for Leadership in Applied Health Research and Care/Applied Research Collaborations from 2008 - present**

| **List of 9 pilot NIHR Collaborations for Leadership in Applied Health Research and Care (CLAHRC) 2008-2013** |
| --- |
| NIHR CLAHRC Birmingham and Black Country  NIHR CLAHRC East of England  NIHR CLAHRC Greater Manchester  NIHR CLAHRC Leicester Northamptonshire and Rutland  NIHR CLAHRC Nottingham Derbyshire and Lincolnshire  NIHR CLAHRC Peninsula (PenCLAHRC)  NIHR CLAHRC Northwest London  NIHR CLAHRC Leeds York Bradford  NIHR CLAHRC South Yorkshire |
| **List of 13 NIHR Collaborations for Leadership in Applied Health Research and Care 2013-2019** |
| NIHR CLAHRC East of England  NIHR CLAHRC East Midlands  NIHR CLAHRC Greater Manchester  NIHR CLAHRC North Thames  NIHR CLAHRC North West Coast  NIHR CLAHRC Northwest London  NIHR CLAHRC Oxford  NIHR CLAHRC South London  NIHR CLAHRC South West Peninsula  NIHR CLAHRC Wessex  NIHR CLAHRC West  NIHR CLAHRC West Midlands  NIHR CLAHRC Yorkshire and Humber |
| **List of 15 NIHR Applied Research Collaborations (ARC) 2019-2024** |
| NIHR ARC East of England  NIHR ARC East Midlands  NIHR ARC Greater Manchester  NIHR ARC Kent, Surrey and Sussex  NIHR ARC North East and North Cumbria  NIHR ARC North Thames  NIHR ARC Northwest London  NIHR ARC North West Coast  NIHR ARC Oxford and Thames Valley  NIHR ARC South London  NIHR ARC South West Peninsula  NIHR ARC Wessex  NIHR ARC West  NIHR ARC West Midlands  NIHR ARC Yorkshire and Humber |

**Appendix 2: Research Excellence Framework impact case studies proforma**

| **REF impact case study URL** |  |
| --- | --- |
| **Panel** |  |
| **Unit of Assessment** |  |
| **Name of Higher Education Institution** | If this is a joint submission add both  Pre-1992 University  Post-1992 University  Russell Group University |
| **Title of case study** |  |
| **For REF 2021: is this a continuing impact case study?** |  |
| **How is this linked to a CLAHRC?** | Is a CLAHRC mentioned directly  Not mentioned directly but verified or data source(s) indicate linked to a CLAHRC |
| **What was CLAHRC’s involvement?** | Contributed to impact  Currently supporting research  Contributed to impact and currently supporting research  Unclear/not specified |
| **From what period is CLAHRC support noted** | If available |
| **What role did the CLAHRC play?** | If specified describe  Not clear/unknown from case study |
| **Who are the main research funders** | If possible to extract, for example:  National Institute for Health and Care Research  Research Councils  Industry  UK charity  Public sector  European Union  Other international  Other  Not specified |
| **Is the case study based on:** | If possible to extract:  The work of one researcher, or the work of a team or the work of several teams |
| **Is the case study based on:** | If possible to extract:   - Small number of different studies (up to three) - Large number of studies (3 plus) |
| **What is the indicative timescale between research and impact?** | - Add date that the research period started - Add date that the impact period started - Add timescale between research commencing and the impact |
| **Impact Category** | Assign using categories from the REF database  Political  Legal  Health  Cultural  Technological  Societal  Economic  Environmental |
| **What is the extent of the impact?** | Local/national/international |
| **Is there evidence of any collaborative work** | For example with:  Higher Education Institutions  Industry  NHS  Government  Voluntary and community sector  International  Public sector/social care |
| **Is there any evidence of coproduction with user participants and engagement** | For example with:  Service users  Patients  Members of the public |
| **What types of evidence are used to demonstrate impact** | Record types of evidence referred to:  Activity (conference/workshop)  Article  Award  Intellectual property  Legal  Media  Report  Testimonial  Other (e.g. toolkits) |
| **In-depth analysis of impact case studies** | |
| **Brief outline of the context** (what was the problem/need/gap) |  |
| **What was the intervention** (if applicable) |  |
| **Brief outline of what impact is claimed** |  |
| **Did the case study generate any actionable tool (s)** |  |
| **What routes/mechanisms are associated with the impact?** | This section is based on interpretive judgement, for example:  Links with policymakers  Membership of related groups/committees (guidelines, advisory positions)  Development and delivery of training  Production of resources to support use  Patient and public involvement  Links with clinicians  Media engagement  International collaboration  Nothing mentioned |
| **Any other observations** |  |
